# Multi-Scale Mixed Attention Network for CT and MRI Image Fusion

**DOI:** 10.3390/e24060843

**Published:** 2022-06-19

**Authors:** Yang Liu, Binyu Yan, Rongzhu Zhang, Kai Liu, Gwanggil Jeon, Xiaoming Yang

**Affiliations:** 1 College of Electronics and Information Engineering, Sichuan University, Chengdu 610064, China; lyang@stu.scu.edu.cn (Y.L.); yby@scu.edu.cn (B.Y.); zhang_rz@scu.edu.cn (R.Z.); 2 College of Electrical Engineering, Sichuan University, Chengdu 610064, China; kailiu@scu.edu.cn; 3 Department of Embedded Systems Engineering, Incheon National University, Incheon 22012, Korea

**Keywords:** convolutional neural network, image fusion, attention, visual saliency

## Abstract

Recently, the rapid development of the Internet of Things has contributed to the generation of telemedicine. However, online diagnoses by doctors require the analyses of multiple multi-modal medical images, which are inconvenient and inefficient. Multi-modal medical image fusion is proposed to solve this problem. Due to its outstanding feature extraction and representation capabilities, convolutional neural networks (CNNs) have been widely used in medical image fusion. However, most existing CNN-based medical image fusion methods calculate their weight maps by a simple weighted average strategy, which weakens the quality of fused images due to the effect of inessential information. In this paper, we propose a CNN-based CT and MRI image fusion method (MMAN), which adopts a visual saliency-based strategy to preserve more useful information. Firstly, a multi-scale mixed attention block is designed to extract features. This block can gather more helpful information and refine the extracted features both in the channel and spatial levels. Then, a visual saliency-based fusion strategy is used to fuse the feature maps. Finally, the fused image can be obtained via reconstruction blocks. The experimental results of our method preserve more textual details, clearer edge information and higher contrast when compared to other state-of-the-art methods.

## 1. Introduction

Since the 21st century, the Internet of Things (IoT) technology has developed rapidly and has been used extensively in the medical field. Many advanced medical technologies rely on the Internet of Things technology, such as medical details, medical process digitisation, material management simulation, and disease diagnosis [1,2,3]. However, the condition of medical care has been increasingly unbalanced. In some remote areas, people have to go to other developed cities to get better medical treatment. To address this issue, telemedicine is proposed. By analyzing medical images, experienced doctors can diagnose diseases online. Medical images have long been used in a variety of clinical areas, including treatment planning, surgical navigation and diagnosis [4]. However, medical images captured from different sensors, on the other hand, might provide distinct kinds of information due to the diversity of imaging techniques [5]. For example, magnetic resonance imaging (MRI) images can offer high-resolution anatomical information for soft tissues and computed tomography (CT) images can accurately detect dense structures such as implants and bones [6]. CT and MRI image can complement their deficient information to obtain a high-resolution structural information image, which is used to diagnose tumours in people with head and neck cancer who are in the early stages [6]. Therefore, during the online diagnostic process, physicians often need to analyze the medical images of these two different modalities separately [7], which leads to inconvenience and inefficiency. In order to help doctors complete telemedicine more easily and effectively, multi-modal medical image fusion is proposed [8].

Traditional methods and deep learning (DL)-based methods are the two types of image fusion. Traditional methods are mostly done at the pixel level, which consists of the spatial domain (SD)-based methods and transform domain (TD)-based method [8]. SD-based methods generally use pixel-based fusion algorithms to compute the relationship between source images in the spatial domain, such as high-pass filter (HPF) [9], principal component analysis (PCA) [10], intensity-hue-saturation (IHS) [11] and brovey transform (BT) [12]. SD methods are simple and easy to implement with low time complexity, but the spectral distortion of the images, which produced by those methods, is more serious. Due to this defect, traditional multi-modal medical image fusion is always based on TD-based methods. TD-based methods usually consist of three steps: source image decomposition, feature fusion and fused image reconstruction. The decomposition and fusion strategies are the core of TD-based methods. For the diversity of multi-modal imaging mechanisms, most TD-based medical image fusion methods selected multi-scale transform (MST) as the strategy of decomposition. The MST-based methods can be divided into three types: wavelet-based transform (e.g., discrete wavelet transform (DWT) [13] and dual-tree complex wavelet transform (DTCWT) [14]), multi-scale geometric analysis (e.g., nonsubsampled shearlet transform (NSST) [15] and nonsubsampled contourlet transform (NSCT) [16]) and pyramid-based transform (e.g., ratio of low-pass pyramid (RP) [17] and Laplacian pyramid (LP) [18]). The MST-based methods usually decompose source images into multiple detail layers and a base layer. However, in the MST-based fusion methods, the global “variance rule” and “averaging rule” are the most popular fusion rules utilized to fuse the base information and a “weighted-average” or “choose-max” fusion rule is often used in the detail information fusion. Due to a variety of issues such as differences in source pixel intensities, noise and misregistration, this kind of fusion strategy is typically not particularly robust [19]. To enhance the fusion performance, many mixed fusion methods are proposed, such as LP-CSR [20] and NSST-PAPCNN [7]. On the basis of the MST-based methods, these mixed methods introduce new fusion strategies to fuse the base layer. However, designing an ideal fusion strategy is very hard and the individual fusion strategies without a strong association may greatly limit the performance of the methods.

The DL-based methods can be divided into two types: generative adversarial network (GAN)-based methods and convolutional neural network (CNN)-based methods [21]. As for the CNN-based type, Liu et al. [22] found that CNN-based architecture has outstanding feature extraction and representation capability. To fuse multi-focus images, they proposed a CNN-based method. They just used a simple CNN architecture and achieved a very good result. Later, they proposed a novel medical image fusion method [19] to overcome the difficulty in designing a robust fusion strategy. In this method, they employed a siamese network to extract source image features and then designed a new fusion strategy, which applied a multi-scale method based on pyramid [23] to improve visual effect and adopted a local similarity-based fusion strategy [24] to fuse deconstructed features. Prabhakar et al. proposed a CNN-based fusion method named as DeepFuse [25] for the exposure image fusion task. They introduced a new network based on an encoder-decoder architecture. Base on DeepFuse [25], Li et al. presented DenseFuse [26], which had an encoder-decoder architecture. Different from the “choose-max” rule of DeepFuse, a new strategy that mixed soft-max and $l_{1}$-norm rule was utilized to merge extracted features. For the GAN-based type, Ma et al. [27] first proposed a GAN-based architecture, termed FusionGAN, to enable the fusion of infrared and visible images. This method avoided problems of time complexities and artificial effects associated with the manual design of fusion rules. The next year, they proposed a new fusion method named DDcGAN [28]. In this method, A dual-discriminator architecture was proposed for multi-resolution image fusion. However, they didn’t highlight recognisable items and their fusion images lost considerable detail. Generally, without too many arbitrarily devised decomposition strategies and fusion rules, the DL-based approach may produce good results. However, their fusion results had a low contrast ratio because they could not highlight significant objects while preserving background information.

Inspired by previous works, we proposed a CNN-based fusion method based on the encoder-decoder architecture to fuse CT and MRI images. Due to the diversity of multi-modal imaging mechanisms, a multi-scale network is employed to extract more crucial features in the encoder block. To improve the fusion effect, we design a mixed attention block to refine the extracted features in the encoder block. To fuse features more accurately, a saliency detection strategy is introduced to highlight useful information in CT images and then merge the features of two source images.

The primary contributions of this work are summarized as follows:

(1) We propose a multi-scale mixed attention block (MMAB). To obtain more information, we adopt a multi-scale feature extraction structure. Furthermore, in order to preserve more useful features, an attention block elaborately mixes spatial and channel attention is introduced. Extensive experiments have demonstrated the superiority of our method.
(2) A novel fusion method is introduced to achieve superior medical image fusion. This method employs visual saliency to highlight useful features. In this way, spatial details and texture features are aggregated to improve performance significantly.

The rest of this paper is arranged as follows: Section 2 briefly reviews related attentions and fusion strategies. Section 3 introduces our proposed method in detail. Section 4 shows the experiments and results. Finally, the conclusions of our paper are drawn in Section 5.

## 2. Related Work

### 2.1. Attention

In recent years, convolutional neural networks (CNNs) have been widely used in the field of image processing for their superior ability to extract information. However, there are so many different tasks in image processing and the required image features are not all the same. The CNNs cannot efficiently change the feature that focuses on various image processing tasks. Larochelle et al. [29] studied the human visual attention module and found that when people look at a picture, they tended to see images piece by piece in a different order rather than see the whole picture first. Base on this conclusion, they proposed an attention module to classify image features into different attention levels and increased the proportion of high attention level features to cope with different image processing tasks. Attention modules had been widely concerned. More and more attention modules were proposed to solve various image processing tasks. Hu et al. introduced a compact block named Squeeze-and-Excitation block (SE) [30] to utilize the relationship between channels. In the SE, they used a global average-pooling which was called squeeze part to compute the attention of different channels. Then, they captured the channel attention maps by an excitation part. However, they only computed the channel-wise attention which described what to focus on and ignored the spatial attention which described where to focus on. Based on SE [30], Roy et al. proposed a spatial attention module based on SE block. They formed a mixed attention module scSE [31] by combining channel-wise attention (CA) and spatial attention (SA) module in parallel for semantic segmentation tasks. Nevertheless, both SE and scSE only took advantage of the average-pooling features and missed the max-pooling features. Woo et al. noticed this issue and proposed a dual-pooling attention module called as CBAM [32] for image classification tasks. They changed the pooling in CA and SA to dual-pooling that calculated the maxi-pooling features and the average-pooling features at the same time. Different from scSE, they arranged CA and SA as sequential channel-spatial.

It is obvious that attention modules are clearly capable of delivering significant benefits in a variety of visual tasks with a slight computational burden. Furthermore, rather than preserve all information, the goal of image fusion is to combine useful features. Therefore, it is essential to design an attention module appropriate for medical image fusion. Inspired by previous works, we propose a dual-pooling attention module that connects CA and SA in parallel.

### 2.2. Fusion Strategy

Many feature fusion strategies have been proposed over the years for image fusion. However, most strategies calculate the weights, either local or pixel, of each extracted feature. The fused image is a weighted sum of the input images.

Burt et al. [24] proposed a fusion method based on the laplacian pyramid decomposition of the image. They performed a fusion strategy based on the saliency and matching of the extracted features. This strategy used region energy to denote saliency and then took the maximum value of saliency or weighted value according to the matching degree of the input images. Li et al. [13] designed a new fusion strategy, which chose max with consistency check. This consistency was determined by the number of pixels with greater absolute value in a region. For example, in the region, when the number of pixels with a larger absolute value is more in image A than in image B, the pixel value of the central pixel point in the region of image A is chosen as the fused pixel value of that point.

The above fusion strategies are traditional methods. They rely on hand-made features for image fusion. Those traditional strategies are not robust because they should change parameters to adapt to different input conditions, e.g., linear and nonlinear exposures, and the filter size depends on the image size. To overcome the issue, Ram et al. [25] designed a CNN-based network to fuse images, named DeepFuse. An encoder-decoder network was used for features extraction and reconstruction. For features fusion, they just added up the extracted features of the two source images. Similar to DeepFuse, DenseFuse [26] also utilized the encoder-decoder network structure. However, DenseFuse found the addition strategy in [25] was very rough to select salient features. Then, they employed a fusion strategy that mixed soft-max and $l_{1}$-norm rule.

Due to the specificity of multi-model medical images, neither addition strategy one nor $l_{1}$-norm strategy could achieve good results. For multi-model medical image fusion, A novel strategy [7] in nonsubsampled shearlet transform (NSST) domain was proposed. To fuse the detail information accurately, a parameter-adaptive pulse coupled neural network (PA-PCNN) [33] was introduced.

**Figure 1:**
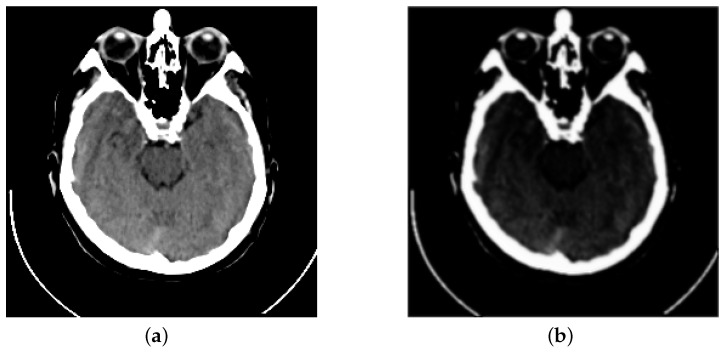
Example of a CT image and its visual saliency map. (**a**) The source CT image of “Acute stroke speech arrest-3”. (**b**) The visual saliency map of this CT image.

Inspired by previous works, we propose a fusion strategy, which is based on the visual saliency-based method (VSM) [34]. We use VSM to calculate the weight maps of computed tomography (CT) images. Then, based on these CT image weight maps we obtain the weight maps of magnetic resonance imaging (MRI) images. Figure 1 presents the visual saliency map of CT image on the image pair “Acute stroke speech arrest-3”. It is obviously observed that VSM of CT can remain the features of dense structures such as implants and bones. Meanwhile, the low-resolution soft tissues are reduced.

### 2.3. Convolutional Neural Networks

The first Convolutional neural network (CNN) is LeNet-5, which was proposed by Yann Lecun et al. [35]. They built the prototype of CNN. However, LeNet-5 was not able to achieve good results at that time. It was not considered seriously.

In 2012, Hinton et al. [36] proposed the AlexNet network for image classification and won the image classification competition that year. Since then, the CNN network has received a lot of attention.

Zeiler et al. [37] made a slight modification to AlexNet and proposed ZF-Net, which helped them win the 2013 image classification competition.

Simonyan et al. [38] proposed the VGG-Nets. They achieved good results by adding more layers to the network. However, VGG-Nets lost to GoogLeNet [39] in the 2014 image classification competition. GoogLeNet introduced the inception structure instead of the traditional convolution operation. This was the first multi-scale CNN.

He et al. [40] proposed the ResNet. They proposed Residual learning to solve the CNN degradation problem and deepened the network depth to 152 layers, which are 8×deeper than VGG-Nets. This is a milestone in the development of CNNs. Almost CNNs have been based on ResNet in the past years.

Huang et al [41] proposed DenseNet, which was based on ResNet. They introduced dense connections to enable feature reuse. Based on ResNet, Gao et al. [42] proposed a multi-scale backbone architecture named as the Res2Net. Res2Net represented multi-scale features at a finer granularity level and increased the perceptual field of each network layer.

Considering the complexity of medical images, we chose Res2Net as our based network. Then, dense connnections were used to improve feature utilisation. We designed a multi-scale CNN, which would be introduced in Section 3.1.

## 3. Proposed Method

**Figure 2:**
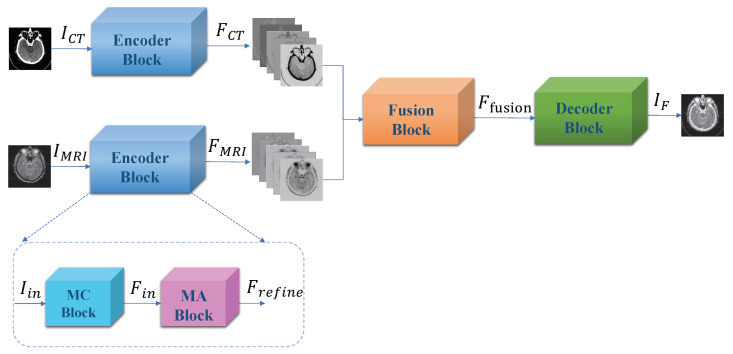
Overall framework of our Muti-scale Mixed Attention Network.

In the section, our multi-scale mixed attention medical image fusion method is introduced detailedly. Figure 2 shows general framework of our proposed computed tomography(CT) and magnetic resonance imaging(MRI) image fusion method. This method mainly consists of three parts: Encoder Block, Fusion Block and Decoder Block. Note that there are two Encoder Blocks in our framework shown in Figure 2, which consist of Multi-scale Convolution Blocks (MC Block) and Mixed Attention Blocks (MA Block). To obtain representative features of source images, these two Encoder Blocks have the same structure and share the same weights. Let us denote $I_{CT}$ and $I_{MRI}$ as the input of Encoder Block. Therefore, we can represent the output of Encoder Block as

(1) $$F_{CT}=f_{En}\left(I_{CT}\right),F_{MRI}=f_{En}\left(I_{MRI}\right),$$

where $f_{En}\left(\cdot \right)$ represents the feature extraction by Encoder Block. $F_{CT}$ and $F_{MRI}$ is the output of the Encoder Block. Then, $F_{CT}$ and $F_{MRI}$ is used for image fusion with our fusion method. This operation can be expressed as

(2) $$F_{fusion}=f_{fusion}\left(I_{CT},F_{MRI}\right),$$

where $f_{fusion}\left(\cdot \right)$ denotes our proposed medical fusion method. $F_{fusion}$ represents the output of fusion method. Then, the fused features are used as input to the Decoder Block to reconstruct the fused image. The fused image can be formulated as

(3) $$I_{F}=f_{De}\left(F_{fusion}\right),$$

where $f_{De}\left(\cdot \right)$ stands for the image reconstruction operation of the Decoder Block.

### 3.1. Encoder Block

**Figure 3:**
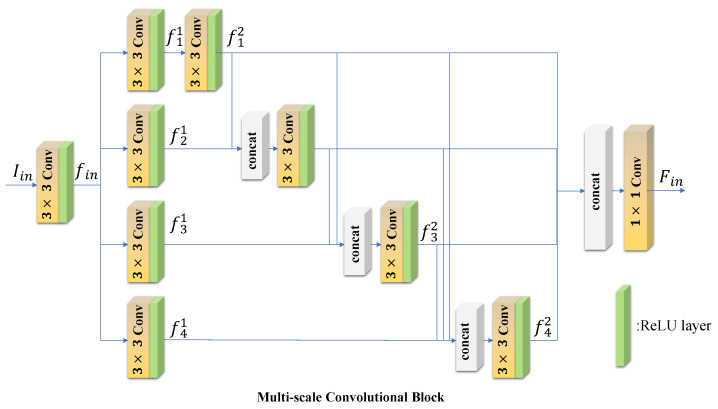
Structure of our MC Block.

Our proposed Encoder Block, which is shown in Figure 2, combines two parts: Multi-scale Convolutional Block (MC Block) and Mixed Attention Block. The first part is MC Block, which is used to extract image features. Image feature extraction plays an essential role in image fusion. CNNs are always employed to obtain deep representations from source images. Res2net [42] is a CNN with powerful feature extraction capability. Inspired by Res2net, we designed a four-branch feature extraction block to extract image features at different scales, which is shown in Figure 3. Our MC Block first uses a 3×3 convolution layer to extract shallow feature maps. Let us denote $I_{in}$ as the input of MC Block. We can represent the shallow feature maps as

(4) $$f_{in}=\rho \left(I_{in}\right),$$

where ρ(·) denotes the operation of the 3×3 convolution layer. $f_{in}$ is then used for the deep feature extraction. Our MC Block designs the deep feature extraction by constructing four hierarchical branches, which can increase the perceptual field. Each branch consists of two 3×3 convolution layers. Different from Res2net, our MC Block uses concatenations [41] to increase the correlation between each branch by combining the second convolutional output of the first few branches with the first convolutional output of the latter branch as the second convolutional input of the latter branch. The operation can be expressed as

(5) $$f_{1}^{1}=\rho \left(f_{in}\right),f_{2}^{1}=\rho \left(f_{in}\right),f_{3}^{1}=\rho \left(f_{in}\right),f_{4}^{1}=\rho \left(f_{in}\right),$$

(6) $$\begin{array}{c} \begin{array}{cc} f_{1}^{2} & =\rho \left(f_{1}^{1}\right),\\ f_{2}^{2} & =\rho \left([f_{1}^{2},f_{2}^{1}]\right),\\ f_{3}^{2} & =\rho \left([f_{1}^{2},f_{2}^{2},f_{3}^{1}]\right),\\ f_{4}^{2} & =\rho \left([f_{1}^{2},f_{2}^{2},f_{3}^{2},f_{4}^{1}]\right), \end{array} \end{array}$$

where $f_{1}^{1}$, $f_{2}^{1}$, $f_{3}^{1}$, $f_{4}^{1}$ denote the output of the first convolution layer at each branch. [$f_{1}^{2}$, $f_{2}^{1}$], [$f_{1}^{2}$, $f_{2}^{2}$, $f_{3}^{1}$], [$f_{1}^{2}$, $f_{2}^{2}$, $f_{3}^{1}$], [$f_{1}^{2}$, $f_{2}^{2}$, $f_{3}^{2}$, $f_{4}^{1}$] denote the concatenations of all features acquired from the previous branch. Furthermore, $f_{1}^{2}$, $f_{2}^{2}$, $f_{3}^{2}$, $f_{4}^{2}$ represent the output of the second convolution layer at each branch. Then, the output feature maps of the four branch are concatenated as the imput of 1×1 convolution layer to integrate multi-scale features. This formulation is defined as follows:

(7) $$\begin{array}{c} F_{in}=\alpha \left([f_{1}^{2},f_{2}^{2},f_{3}^{2},f_{4}^{2}]\right), \end{array}$$

where α(·) denotes the operation of the 1×1 convolution layer. $F_{in}$ is then utilized for the MA Block to refine the informaiton at spatial and channel-wise levels.

**Figure 4:**
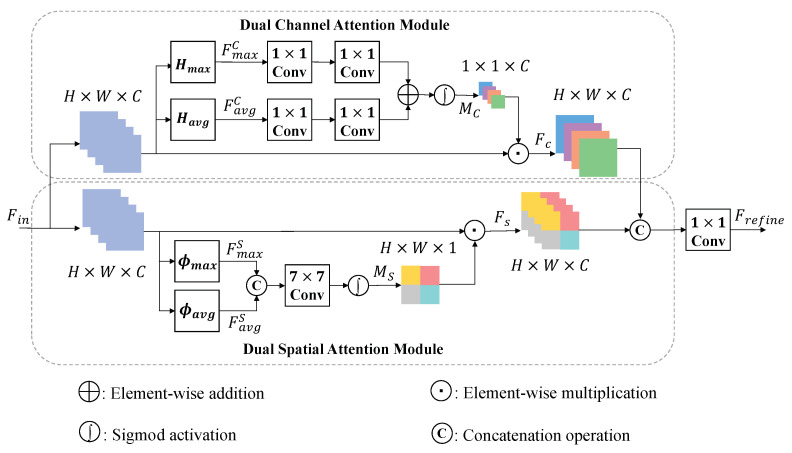
Diagram of our MA Block.

Figure 4 shows the diagram of our proposed MA Block. The refined feature maps are calculated from the input feature maps $F_{in}$ by parallel CA and SA, respectively. Then, the two refined feature maps are concatenated and input into a 1×1 convolution to get the final feature maps $F_{refine}$.

In the part of CA, there are two parts. The first one is spatial squeeze module and another is channel excitation module. The spatial sequeeze means we want to pour the input feature maps $F_{in}\in R^{H\times W\times C}$ as a 1×1×C weight vector, which shows ‘what’ to focus on. Therefore, $F_{in}\in R^{H\times W\times C}$ is first imported into a dual-pooling to calculate the max-pooling $F_{max}^{C}\in R^{1\times 1\times C}$ and the average-pooling $F_{avg}^{C}\in R^{H\times W\times 1}$ separately, defined as

(8) $$\begin{array}{c} \begin{array}{c} F_{max}^{C}=H_{max}\left(F_{in}\right),\\ F_{avg}^{c}=H_{avg}\left(F_{in}\right), \end{array} \end{array}$$

where $H_{max}\left(\cdot \right)$ and $H_{avg}\left(\cdot \right)$ mean the operations of spatial max-pooling and spatial average-pooling. Then, $F_{max}^{C}\in R^{1\times 1\times C}$ and $F_{avg}^{C}\in R^{1\times 1\times C}$ enter the excitation module to generate the refined feature maps $F_{C}$. The excitaition module consists of a $1\times 1\times \frac{c}{16}$ convolution layer, a 1×1×C convolution layer, an element-wise addition operation, a sigmod activation and an element-wise multiplication operation. It can be represented as

(9) $$\begin{array}{c} M_{C}=\sigma \left(F_{C1}\left(\delta \left(F_{C2}\left(F_{max}^{C}\right)\right)\right)⊕F_{C1}\left(\delta \left(F_{C2}\left(F_{avg}^{C}\right)\right)\right)\right), \end{array}$$

where ⊕ defines as the element-wise addition operation. $F_{C1}\left(\cdot \right)$ and $F_{C2}\left(\cdot \right)$ represents convolution layers. δ(·) denotes rectified linear unit (ReLU) and σ(·) is the sigmod activation. $M_{C}\in R^{1\times 1\times C}$ is the output weight vector of CA. Then, the final refined features of the ath channel ($F_{C}^{a}$) is obtained by rescaling the input ($F_{in}^{a}$)

(10) $$\begin{array}{c} F_{C}^{a}=F_{in}^{a}\cdot M_{C}^{a}, \end{array}$$

where $M_{C}^{a}$ is the ath weight in the cth channel. · refers to the element-wise multiplication operation.

Similar to CA, the part of SA consists of channel squeeze module and spatial excitation module. The channel squeeze module is used to squeeze the input feature maps $F_{in}\in R^{H\times W\times C}$ as a H×W×1 weight map, which shows ‘where’ to focus on. In the channel squeeze module, the $F_{in}\in R^{H\times W\times C}$ input into a dual-pooling to obtain $F_{max}^{S}\in R^{H\times W\times 1}$ and $F_{avg}^{S}\in R^{H\times W\times 1}$. This formulation is defined as follows:

(11) $$\begin{array}{c} \begin{array}{c} F_{max}^{S}=\phi_{max}\left(F_{in}\right),\\ F_{avg}^{S}=\phi_{avg}\left(F_{in}\right), \end{array} \end{array}$$

where $\phi_{max}\left(\cdot \right)$ and $\phi_{avg}\left(\cdot \right)$ denote the operation of channel max-pooling and channel avg-pooling. $F_{max}^{S}$ and $F_{avg}^{S}$ are then used to obtain the final refined features map $F_{S}$ with a spatial excitation module. Before that, we should acquire the spatial weight map $M_{S}\in R^{H\times W\times 1}$ by a 7×7×C convolution layer and a sigmod activation. This procedure can be expresssed as

(12) $$\begin{array}{c} M_{S}=\sigma \left(F_{C3}\left([F_{max}^{S},F_{avg}^{S}]\right)\right), \end{array}$$

where $F_{C3}\left(\cdot \right)$ denotes 7×7×C convolution layer. $\left[F_{max}^{S},F_{avg}^{S}\right]$ is the concatenation of $F_{max}^{S}$ and $F_{avg}^{S}$. Then, the final refined features of the ath channel ($F_{S}^{a}$) is obtained by rescaling the input ($F_{in}^{a}$)

(13) $$\begin{array}{c} F_{S}^{a}=F_{in}^{a}\cdot M_{S}, \end{array}$$

where · represet the element-wise multiplication operation.

Then the two refined feature maps of CA and SA are concated and input into a 1×1×C convolution layer. Therefore, we can get the final output ($F_{refine}$) of our MA as

(14) $$\begin{array}{c} F_{refine}=F_{C4}\left([F_{C},F_{S}]\right), \end{array}$$

where $F_{C4}\left(\cdot \right)$ is the 1×1×C convolution layer operation. $\left[F_{C},F_{S}\right]$ denotes the concatenation of the output of the two attention modules.

### 3.2. Fusion Block

After the Encoder Block, we obtain the feature maps $F_{CT}$ and $F_{MRI}$, which are represented as the unique information of CT and MRI images. It is difficult to adequately combine CT and MRI images since they are acquired by distinct sensors. As we know, CT images can provide the more precise features of dense structures such as implants and bones. However, it does not achieve good performance in soft tissue, while MRI image does the opposite. In image fusion, selecting weight maps for feature fusion is crucial. If the features of the two source photos are simply added together, there will be many significant features lost and textures blurred. We introduce a visual saliency-based method (VSM) to our fusion strategy in order to better fuse these multi-modal features. The visual saliency value is denoted as $VSM_{I_{n}}$ and it shows the significance of pixel $I_{n}$ in the image, which is represented as [34]:

(15) $$\begin{array}{cc} f_{VSM}\left(I_{n}\right) & =∥I_{n}-I_{0}∥+∥I_{n}-I_{2}∥+\ldots +∥I_{n}-I_{N}∥, \end{array}$$

(16) $$\begin{array}{cc} \; & =\sum_{m=0}^{N}M\left(m\right)∥I_{n}-I_{m}∥, \end{array}$$

where *N* denotes the level of intensity, which is 255 in gray images. M(m) is the frequency of $I_{m}$, while m represents the intensity value. The histogram of the image is used to reduce the computational complexity in VSM, which may be achieved in O(N) time order.

**Figure 5:**
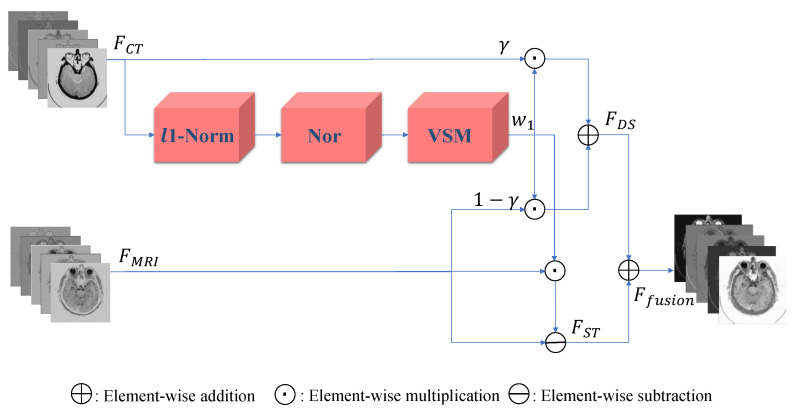
Diagram of our Fusion Block.

As shown in Figure 5, A fusion strategy based on VSM is proposed. Our fusion strategy computes the weight maps based on the refined feature from CT images. To quantify activity level, we first employ the l1-Norm strategy, which is defined as the initial weight map. By doing that, we can express source image features. The initial weight map $IWM_{CT}\in R^{H\times W\times 1}$ is computed as follows [34]:

(17) $$IWM_{CT}\left(x,y\right)={∥F_{CT}^{a}\left(x,y\right)∥}_{1},$$

where $F_{CT}^{a}\left(x,y\right)$ denotes the features from CT images extracted by Encoder Block and a refers to the channel number. $IWM_{CT}\left(x,y\right)$ represents the initial weight at position (x,y) and then is refined using the Nor(·) function. To make the visual saliency map more representative, the Nor(·) function increases the distance of saliency values [34]:

(18) $$w_{Nor}=\frac{IWM_{CT}-\min\left(IWM_{CT}\right)}{\max\left(IWM_{CT}\right)-\min\left(IWM_{CT}\right)},$$

Then the weight map $w_{1}$ is computed by

(19) $$w_{1}=f_{VSM}\left(w_{Nor}\right),$$

where $w_{1}$ represents the part of dense structures on CT image. For dense structures, the features of MRI images can also be useful for diagnosing conditions such as osteoporosis. Therefore, we designs the feature of dense structures($F_{DS}$) as

(20) $$F_{DS}^{a}=\gamma \left(w_{1}\cdot F_{CT}^{a}\right)+\left(1-\gamma \right)\left(w_{1}\cdot F_{MRI}^{a}\right),$$

where · denotes the matrix-wise multiplication, a represents the ath channel. γ refers to the weight of CT image features at the dense structures part of fused image. Another part of features are all extracted from the MRI image, which is expressed as

(21) $$F_{ST}^{a}=\left(1-w_{1}\right)\cdot F_{MRI}^{a},$$

Then the final fused features $F_{fusion}$ are the sum of $F_{DS}$ and $F_{ST}$, denoted as

(22) $$F_{fusion}=F_{DS}+F_{ST},$$

### 3.3. Decoder Block

**Figure 6:**
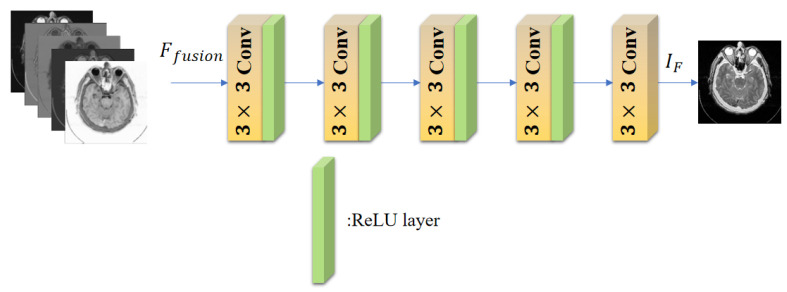
Diagram of our Decoder Block.

The last part of our proposed method is Decoder Block, which is utilized to recover the fused image $I_{F}$ from fused features $F_{fusion}$. As illustrated in Figure 6, the Decoder block consists of five common convolution layers of 3×3 kernels. The number of channels is set from 240 to 1 layer by layer. For activation functions, the previous four layers are ReLU, while the final layer lacks it. The number of convolution layers is variable. More layers can preserve more information but increase the computation burden. We chose the applicable image reconstruction setting in consideration of fusion performance and efficiency.

### 3.4. Training Details

**Figure 7:**
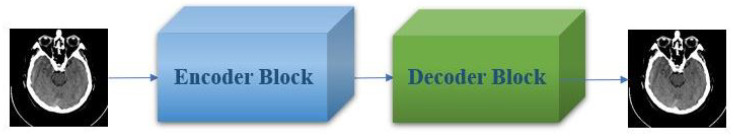
General framework of our training phase.

Encoded Block and Decoder Block are employed, during the training phase, to construct the backbone network. specifically, as it is shown in Figure 7, we remove the Fusion Block. To fully utilize the multi-scale information for matching the source image, the encoder-decoder structure is adopted, which shows outstanding performance in image fusion.

A loss function *L* mixed the gradient loss and the pixel loss is employed to reconstruct the source image more accurately. The gradient loss characterizes the structural information and the pixel loss is used to describe energy and details information [43]. The *L* is set to

(23) $$L=\frac{1}{WH}\left(\mu {∥I_{F}-I_{in\;}∥}_{2}^{2}+\varphi {∥\nabla I_{F}-\nabla I_{in}∥}_{2}^{2}\right),$$

where ∇ denotes the gradient operator, ${∥\cdot ∥}_{2}$ refers to $L_{2}$-norm, and WH stands for the spatial size of source images. μ and φ are variables used to achieve a balance between the two items. $I_{in\;}$ represents the input training image. $I_{F}$ indicates the output image

## 4. Experiments and Results Analysis

### 4.1. Experimental Settings

**Datasets** During training, 561 pairs of CT and MRI images are utilized as our based dataset, which are acquired from Whole Brain Atlas [44]. Then, we choose 400 pairs of CT and MRI images from the based dataset as the training dataset. They are cropped and rotated to augment 4000 training image pairs, which are used to train our encoder-decoder architecture. During the validation process, we select 59 pairs of CT and MRI images from the based dataset. After training, we set the remainder 102 pairs of CT and MRI images from the based dataset as our evaluation datasets.

**Comparative methods** We compare our method to six different image fusion methods in both objective and subjective evaluation to certify its superiority. These methods include ASR [45], GFF [46], LP-SR [47], NSST-PAPCNN [7], CNN [19], DenseFuse [26] and IFCNN [48]. Among them, ASR is base on TD. GFF is based on SD. LP-SR and NSST-PAPCNN are mixed fusion methods. CNN, DensFuse and IFCNN belong to DL-based methods. All the parameter of comparative methods is set as the corresponding papers given by authors.

**Evaluation metrics** We select six commonly used objective evaluation indexes to assess the experimental results, including CC [49], MI [50], NCIE [51], SF [52], PC [53] and SCD [54]. CC, MI, NCIE, PC and SCD describe the correlation between the source image and the fused image. CC denotes the linear correlation. MI means the amount of remained information. NCIE shows the general relationship. PC calculates the preserved salient features. SCD represents the amount of complementary information. SF is only computed from the fused image, which indicates the richness of the texture information. The better the fusion approach performs, the greater the values of these six metrics in a fused image.

**Implementation details** In our training phase, Adam [55] is our parameter optimizer and batch size is 16. The learning rate is initialed as 0.0001 and epoch number is 1000. NVIDIA TITAN Xp (GPU), Intel Xeon (R) E5-2680 v4 @ 2.40 GHz × 56 (CPU) and 128 GB RAM (Memory) are used to train our proposed framework. The network architecture is programmed on the Pytorch 1.9.0.

### 4.2. Comparison with Other Methods

Seven excellent methods and ours are evaluated subjectively and objectively to attest to the advantages of our method. The first part is the subjective assessment.

**Figure 8:**
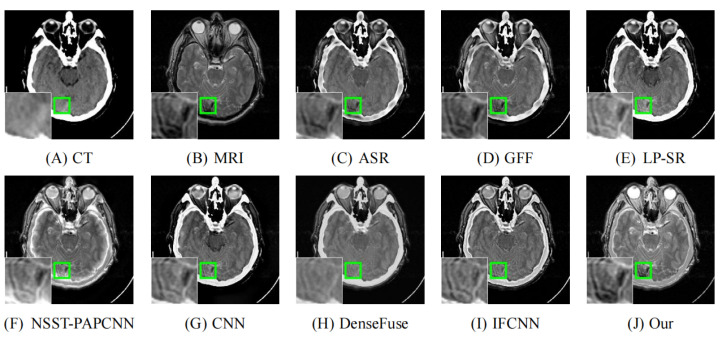
Fusion images of “Acute stroke speech arrest-7” by various methods. From (**A**) to (**J**): CT, MRI, ASR, GFF, LP-SR, NSST-PAPCNN, CNN, DenseFuse, IFCNN, Our proposed method.

**Figure 9:**
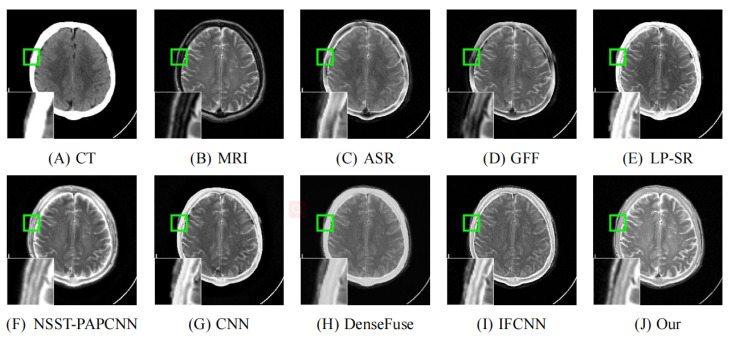
Fusion images of “Acute stroke speech arrest-16” by various methods. From (**A**) to (**J**): CT, MRI, ASR, GFF, LP-SR, NSST-PAPCNN, CNN, DenseFuse, IFCNN, Our proposed method.

**Figure 10:**
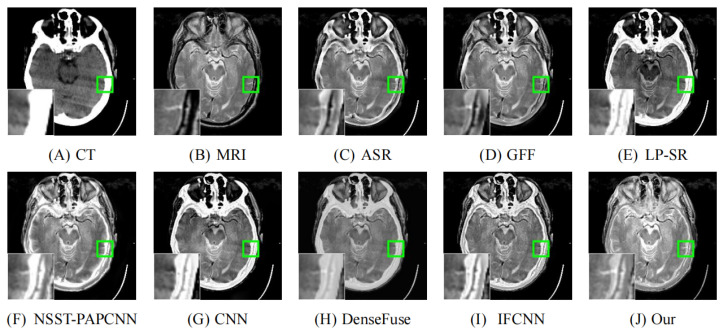
Fusion images of “Meningioma-7” by various methods. From (**A**) to (**J**): CT, MRI, ASR, GFF, LP-SR, NSST-PAPCNN, CNN, DenseFuse, IFCNN, Our proposed method.

**Figure 11:**
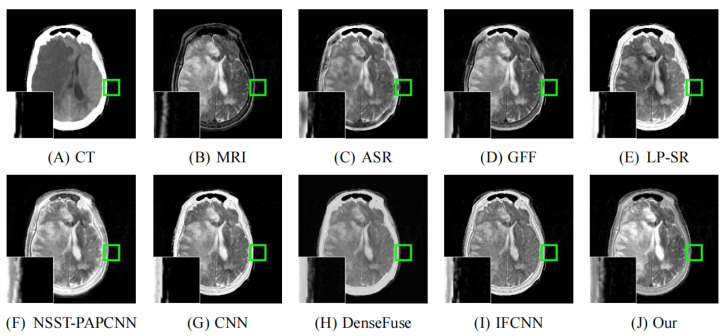
Fusion images of “Fatal stroke-14” by various methods. From (**A**) to (**J**): CT, MRI, ASR, GFF, LP-SR, NSST-PAPCNN, CNN, DenseFuse, IFCNN, Our proposed method.

**Figure 12:**
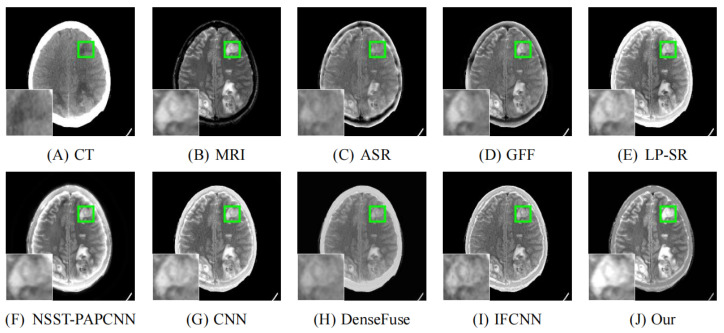
Fusion images of “Sarcoma-17” by various methods. From (**A**) to (**J**): CT, MRI, ASR, GFF, LP-SR, NSST-PAPCNN, CNN, DenseFuse, IFCNN, Our proposed method.

As illustrated in Figure 8, Figure 9, Figure 10, Figure 11 and Figure 12, we select fusion results of five pairs of images as the visual comparison. Among these figures, source images (CT and MRI) are shown in (**A**) and (**B**). (**C**) to (**J**) are fusion results obtained by various fusion methods. Every image has an enlarged subimage in the lower-left corner that is useful for visual assessment.

Although it’s hard to assess the visual effects of these results accurately, significant differences are observed. Figure 8, Figure 9, Figure 10, Figure 11 and Figure 12 show CT images that are high-resolution in dense structures such as bones and implants but low-resolution in soft tissues. MRI images can offer high-resolution edge and anatomical information for soft tissues. However, the other seven methods are affected to varying degrees by the low-resolution soft tissues information of the CT images. It may cause the fused image with fewer details and blurred contours. ASR is a TD-based method that calculates weight maps by some pre-trained dictionaries. The results of ASR have a low contrast ratio and halo effect. As illustrated in Figure 8, the enlarged subimage of ASR has a low contrast ratio and the bone of fused image has a black halo. GFF is a TD-based method. This method decomposes the source images into base layer and detail layer. Then, multiple filters, such as average filter and guided filter, are utilized to merge the features. Thus the contrast is poor in the fused images obtained by GFF. Figure 9 shows that a part of bone in the enlarge subimage is missing. LP-SR and NSST-PAPCNN are mixed fusion methods, which employ MST to decompose the source image and then use different strategies to fuse the high-frequency features and the base features, respectively. LP-SR employs Laplacian pyramid to decompose the source image and then uses the “choose max” rule to fuse the high-frequency features. The multi-scale decomposition and activity level measurements are required for MST-based fused results. This may result in the loss of some details. For example, in Figure 8 and Figure 11, the fused image of LP-SR is more close to the CT image and lose the detail and structure information of MRI image. NSST-PAPCNN employs NSST to decompose the source image and then uses PAPCNN to fuse the detail textures. The soft tissue detail of fused image by NSST-PAPCNN is close to our fusion result, whereas is fuzzier in the edge detail, such as the enlarged part of Figure 11. CNN, DenseFuse and IFCNN are DL-based methods. However, CNN uses LP and GP to fuse the extracted features, which leads to the loss of details from MRI images. Similar to LP-SR, we can also observe the fused image of CNN lose the detail and structure information in the Figure 8 and Figure 11. Similar to our proposed method, DenseFuse uses an encoder-decoder architecture, but the l1−Norm strategy is used in the fusion block. This strategy may make the detail textures of fused images by DenseFuse be smoothed, which can be observed in all the five fused results. Different to our proposed method, IFCNN adds the fusion block to the training phase. It selects elementwise-maximum as the fusion strategy. However, the contrast is lower than our proposed method and the edge detail is not clear enough in the fused result by IFCNN. Overall, the comparison experiment demonstrates our method preserves more textual details and clearer edge information. In addition, due to the higher contrast, our fused images are friendly to human vision.

For objective evaluation of fusion results from different methods, we assess our proposed method as well as the other seven fusion methods on our evaluation datasets. Table 1 shows the average results of six objective fusion evaluation indicators. According to the table, our fused images have the maximum CC, MI, SF, NCIE and SCD. The PC is a little less than the result of GFF. The maximum CC means that our fused images are more linearly correlated with source images. The maximum MI indicates that our fused images retain more information from source images. The maximum SF represents that our fused images are richer in structural and texture information. The maximum NCIE denotes that our method can get more correlated fused images with source images. The maximum SCD means that our method captures more complementary information. Although the PC of GFF is best, it is poor at retaining complementary information. The relatively big PC reveals that our method preserves more salient information. Therefore, our proposed method obtains greater performance and the fused images are closely associated with source images.

### 4.3. The Study of Ablation

#### 4.3.1. Extraction Block

In this subsection, we design two kinds of MC blocks. The Res2 Block (Res2B) is the first. The Res2B is inspired by Res2net and consists of shallow feature extraction and deep feature extraction. The shallow feature extraction is a 3×3 convolution layer. The deep feature extraction has four branches and every branch has two 3×3 convolution layers. We can denote the jth convolution layer’s output of ist branch as $f_{i}^{j}$. For the first branch, the input of $f_{1}^{2}$ is just $f_{1}^{1}$. For the other three branches, the input of the second convolution layer on the ist branch can be expressed as the concatenation of $f_{i-1}^{2}$ and $f_{i}^{1}$. Then, we design Res2 Dense Block (Res2DB) by adding concatenations [41] on the base of Res2B, which is shown in Figure 3. To ensure the rationality of the experiment, we only use the two MC Blocks as the Encoder Block (MA Block is removed). Then, we keep the fusion strategy and Decoder Block same as our proposed method. Table 2 displays the evaluation metrics. All the six evaluation metrics of Res2DB-based method are higher than Res2B and the significantly increased MI means that Res2DB is able to extract more features from source images.

#### 4.3.2. Attention Module

In the subsection, we keep MC Block, fusion strategy and Decoder Block same as our proposed method to test our attention module. In the experiment, we set five different methods, such as Res2D, Res2D and CA, Res2D and SA, Res2D and our proposed attention module. To be consistent with our method, both CA and SA employ dual-pooling to squeeze features. As shown in Table 3, MI, PC, NCIE and SCD of our proposed attention module are the maximum in the five methods. CC and SF of ours is the second maximum. Because our attention is a CA and SA mixed attention. That causes the linear correlation slightly lower than the max of CA and SA. However, other five evaluation metrics are higher than both CA and SA. In general, our attention module is more suitable to preserve useful features.

#### 4.3.3. Fusion Strategy

To illustrate the advantage of our fusion strategy, five strategies are contrasted to our proposed strategy, such as elementwise-average (Avg), elementwise-maximum (Max), elementwise-sum (Sum), l1-Norm and Nuclear. These five strategies are widely used in fusion methods. Due to the simplicity, Avg, Max and Sum are the most popular strategies. DL-method may use the three strategies to merge different kinds of source images. For example, IFCNN uses elementwise-maximum to fuse infrared and visible image. For multi-exposure images, it sets elementwise-average as the fusion strategy. l1-Norm is used in DenseFuse. Nuclear is utilized in MDLatLRR [56]. The evaluation metrics is shown in Table 4. Because Avg, Max and Sum are linear calculations, the CC of their results is the maximum. Except for them, our proposed strategy has the maximum CC. In the rest five metrics, MI, SF, NCIE and SCD are higher than other methods. PC is slightly lower than l1-Norm. It is obvious that our fusion strategy is more superior than the other five strategies.

## 5. Discussion

Multi-modal medical image fusion is important to help doctors complete telemedicine more easily and effectively. CNNs, as we know, are commonly used in the field of image fusion especially towards infrared and visible images. Although significant improvement has been achieved in the above-mentioned fields, it has rarely been adopted in the medical scene. Existing CNN-based medical image fusion methods are not suitable enough for medical image fusion duo to its poor ability for feature refinement [48]. To develop medical image fusion, we designed a CNN utilizing a novel fusion strategy for high-quality CT and MRI image fusion.

Our work had two limitation. One is that the dataset of our work was limited to CT and MRI images. The other was that our work can only fuse two images at once. The assumption was that MRI images can offer high-resolution anatomical information for soft tissues and CT images can accurately detect dense structures such as implants and bones [6]. CT and MRI image can complement their deficient information to obtain a high-resolution structural information image.

Image fusion combined two main parts: features extraction and features fusion. Specifically, the features extraction should extract more suitable features from the source image and the features fusion needed to reserve more useful information. Consistent with previous studies of CNN [19,25,26,42,48] and attention module [29,30,31,32], CNN can extract more information from source images compare to MST. However the features extracted by CNN were broad but not suitable for all image processing tasks. It was essential to employ attention module to features for various tasks. Therefore, we desined a Multi-scale Mixed Attention Network (MMAN) to extract and refine image features. For features fusion, we considered combining the soft tissue features of MRI images and the features of dense structures of CT images, which was different from other CNN fusion methods [25,26,48]. These methods did not specifically design the features fusion module, which just used the “weighted-average” or “choose-max” fusion rule. These rules were good for a general framework for image fusion, but were not accurate for CT and MRI images fusion.

Our work can be divided into three parts: CNN framework design, attention module design and fusion strategy design. As mentioned before, CNNs were rare in medical image fusion, so we learnt CNNs from other fields. Res2net [42] and Densenet [41] were two advanced CNNs. Rest2net added image features at different scales with its multi-scale extraction module. Densenet achieved the full use of shallow information through feature reuse. We combined Res2net and Densenet to propose a Res2DB. Dense mechanism would increase the features extraction ability and the correlation between extracted features and source images through featrue reuse. Then, we designed a ablation experiment to prove it in Section 4.3.1. As shown in Table 2, after adding dense mechanism, all six metrics had improved and MI was significantly increased.

For attention module design, CA and SA were wildly used now. Both of them could highlight critical features to refine features. CA focused on the connection of features between channels. By assigning different weights to the channels, CA was able to increase the weight of useful information at Channel level, which results in the fused image retaining more of the useful information of the source images but lacking the linear correlation. It can be demonstrated by high MI and low CC as Table 3. Different from CA, SA focused on the spatial connection of features. Therefore, it focused on improving linear correlation between the fused image and source images as Table 3 shows. CA and SA had different but important ability to refine features. As is shown in Figure 4, we tried to combine their advantages and design the Mixed Attention. In terms of results, Mixed attention got high MI and middle CC. Because both CA and SA can improve MI, but CA would decrease CC. The other metrics had a little advance due to the increased computational volume.

For various kinds of image fusion, CNN-based image fusion always employed a simple strategy to improve robustness, such as elementwise-average (Avg), elementwise-maximum (Max), elementwise-sum (Sum), l1-Norm and Nuclear. However, we were designing a fusion method only for CT and MRI image fusion. These strategies were too simple to get good results. We considered reducing the weight of the low-resolution soft tissues of the CT image and increase the weight of dense structures. This idea was similar to MCAFusion [57]. MCAFusion employed VSM [34] to extract the visual salient features of infrared and visible images and then fused those features. However, to processing two source images was not suitable for CT and MRI image fusion. We used VSM to extract CT images, as is shown in Figure 5. As is shown in Section 4.3.3, ablation experiments have demonstrated that the use of VSM significantly improved the MI, SCD and SF of fused images, which indicated that VSM retained more correlation information and fused images was more closely related to the source image.

The main contribution of our work can be devided into two pieces. Firstly, we optimized the feature extraction module based on previous work [19,25,26,42,48] and proposed a mixed attention module for CT and MRI image fusion. Good experimental results proved the superiority of our method. Finally, we introduced visual saliency to our fusion strategy. This may offer a new direction of optimization for multi-model medical image fusion.

## 6. Conclusions

This paper proposed an encoder-decoder network-based CT and MRI image fusion method to facilitate telemedicine for doctors. On the one hand, it improved the advanced CNN and attention module. These modules were introduced into medical image fusion field, significantly improving the feature extraction capability. On the other hand, it proposed a VSM-based fusion strategy for CT and MRI image fusion. This strategy provided an idea for future fusion of CT and MRI images. On the Whole Brain Atlas database [44], we used a mixed loss function to train the encoder-decoder network. Firstly, the multi-scale MA block was designed to extract more features at various scales while preserving valuable ones. Then, the extracted features were merged by a visual saliency-based fusion strategy. Finally, a reconstruction network was employed to recover the fused image. The texture features and edge information were well preserved in the fused images of our method. In addition, our method performed excellently across six evaluation metrics. In contrast to seven representative methods, experimental findings showed that our method was superior in terms of visual effect and objective evaluation. Our next challenge is to expend the application scope to fuse any two modalities of medical images, not limit to two CT and MRI images. Furthermore, we are trying to fuse three or more source images at once, not limit to two source images.

## Figures and Tables

**Table 1:** The average objective evaluation indicators comparison between seven excellent fusion methods and our proposed method on our evaluation datasets. Bold font is the best. Underlining font denotes the second best results.

Reference	Method	Evaluation Metrics					
		CC	MI	SF	PC	NCIE	SCD
[45]	ASR	0.7705	3.1663	7.2747	0.3275	0.8073	1.3185
[46]	GFF	0.7731	2.9165	7.3147	**0.4176**	0.8068	1.2891
[47]	LP-SR	0.7592	2.9066	7.2119	0.3419	0.8066	1.4518
[7]	NSST-PAPCNN	0.8008	2.7350	7.8123	0.2797	0.8061	1.4940
[19]	CNN	0.7747	2.8595	7.3321	0.3413	0.8064	1.4558
[26]	DenseFuse	0.8055	3.5654	6.0700	0.3516	0.8086	1.4170
[48]	IFCNN	0.8020	2.9998	7.8717	0.3132	0.8068	1.4890
	Ours	**0.8179**	**4.2488**	**7.8951**	0.3882	**0.8124**	**1.6040**

**Table 2:** The comparison of evaluation metrics on evaluation datasets for the dense mechanism.

Method	CC	MI	SF	PC	NCIE	SCD
Res2B	0.8106	4.0939	7.6834	0.3247	0.8114	1.5485
Res2DB	0.8111	4.1750	7.7203	0.3261	0.8118	1.5545

**Table 3:** The evaluation metrics comparison between different attention modules on evaluation datasets. Bold font is the best. Underlining font denotes the second best results.

Method	CC	MI	SF	PC	NCIE	SCD
Res2D	0.8111	4.1750	**7.7203**	0.3261	0.8118	1.5545
Res2D+CA	0.8108	4.2108	7.6907	0.3300	0.8119	1.5580
Res2D+SA	**0.8125**	4.1930	7.6672	0.3361	0.8118	1.5613
Res2D+Ours	0.8117	**4.2623**	7.6917	**0.3405**	**0.8122**	**1.5621**

**Table 4:** The evaluation metrics comparison between various fusion strategies on evaluation datasets. Bold font is the best. Underlining font denotes the second best results.

Method	CC	MI	SF	PC	NCIE	SCD
Avg	0.8321	3.6557	6.2708	0.2990	0.8091	1.4934
Max	**0.8329**	3.5677	6.2542	0.2973	0.8088	1.4857
Sum	0.8319	3.6477	6.2744	0.2988	0.8091	1.4737
l1-Norm	0.7994	3.6070	6.0722	**0.3650**	0.8087	1.4307
Nuclear	0.8092	3.9211	5.9126	0.3157	0.8099	1.4793
Ours	0.8117	**4.2623**	**7.6917**	0.3405	**0.8122**	**1.5621**

## Data Availability

The dataset of this research are acquired from https://www.med.harvard.edu/aanlib/home.html (accessed on 20 October 2021).
